# Disorders of the Heart

**Published:** 1908-04-25

**Authors:** 


／# The Hospital

A Journal of

The Medical Sciences and Hospital Administration.

New Series. No. 58, Vol. III. [No. 1130, Vol. XLIV.] SATURDAY, APRIL 25, 1908.

### DISORDERS OF THE HEART.

In the third of his Lumleian lectures Sir James Sawyer devoted much attention to what he terms the disorders, as distinguished from the diseases, of the heart. Certainly the common teaching of the schools does not sufficiently emphasise the importance of this large group of cardiopathies, and the neglect results in no little harm. Although students hear in vague terms of functional murmurs and functional arrhythmia, there is no doubt that most of them go out into the world persuaded that a heart-murmur for the most part means a valvular lesion, and arrhythmia a myocardial degeneration except in the case of heavy smokers. This very faulty creed not only leads to the rejection by insurance offices of a certain number of perfectly eligible lives, but results in medical verdicts which consign sound but timid individuals to lives of unnecessary invalidism. The fundamental reason for this insufficient education in the matter of the heart is that functional disturbances of this organ do not lend themselves to the purposes of examination. The student knows that if fate brings him a heart-case in the course of his practical examination it will be one of a small group of organic maladies. He therefore focusses his attention upon the common valvular diseases, and very likely becomes expert enough in the hearing and timing of murmurs. Indeed he is often encouraged to expend a superfluous subtlety in differentiating early, middle, and late diastolic murmurs and such things, until he has the auscultation of the heart at his fingers' ends. But it is not sufficiently brought home to him that, as has been said by a physician long versed in teaching, "any fool can hear a murmur," and that the test of skill is the correctness of his interpretation of what he hears. Living as he does in an atmosphere of organic lesions, he does not truly appreciate the significance of casual remarks upon functional cardiac disorders until he has paid for knowledge at the expense of disagreeable experiences. It is not easy to see how this narrowness of view can be avoided, considering the quantity of knowledge which students of medicine are expected to assimilate, until some alteration is made in the quality of the examinations to which they have to submit themselves. The existing state of affairs certainly calls for remedy. Although experienced physicians at all times have urged the necessity of studying the patient and not his malady, the good advice is far too little observed by the teachers of medicine, and their disciples enter the lists proportionately ill-equipped.

In considering functional disorders, whether of the heart or any other organ, this width of view is indispensable, for functional disorders in bulk are merely indicators of a certain type of mental constitution. "The nervous temperament," says Sir James, in the lecture to which we have alluded, "is the basis of the inorganic maladies of the heart. The recognition of that temperament is the clue to their successful management. In these affections the signs of the patient's disposition interpret the symptoms of his indisposition." These are wise words, for they accent the futility of dealing by chemical or physical means—by drugs, or hydrotherapy, or exercises—with maladies which are temperamental in origin. Worry, as the lecturer justly observed, is the foundation of many cardiac neuroses. Often enough this worry is the indirect result of the functional disorder concerned; yet it perpetuates the disturbance which has given it birth, and can be dispelled by sensible and reassuring advice. It is very true that "worry is a continued form of impatience," and the treatment of such temperamental disabilities must clearly be moral before all things.

Of the physical evidences of functional disturbance, Sir James notes as the commonest an apical systolic murmur associated with excited beating of the heart. This has been called the "souffle de consultation." It is almost invariably limited to the apical region of the heart, and is found apart from any other evidence of a heart-lesion. But there are other murmurs whose mechanism remains unknown but which are compatible with perfect health and with a heart of normal dimensions. For example, there is a systolic murmur heard only during inspiration. This murmur is often widely spread and loud, yet disappears as the emotional excitation of the heart subsides with the growing confidence of the patient. There is a similar, but less intense, systolic murmur which persists during normal breathing, to disappear when the lungs are completely emptied by a forced expiration. There is, of course, the familiar systolic murmur heard at the pulmonary area, a murmur which may be so loud as to discredit the belief that it does not signify an organic lesion, yet persists for years without any corroborative indication of harm. Disturbances of rhythm are so common among healthy subjects as to be negligible, at least in early life, unless there is other evidence of disease. It is a commonplace of practice that those who complain of palpitation seldom show any signs of organic change in the heart, while intermittencies and irregularities in the beating of a diseased heart may pass without comment from the patient unless they be extreme. These anomalies of function, to which many others might be added, give point to the lecturer's assertion that "a heart case is never only a heart case, be it either a functional one or be it an organic one." Even when there exists a definite structural alteration it does not follow that the latter alone is responsible for the symptomatology with which it is associated. "We are too ready to assume," writes Sir Thomas Clifford Allbutt, "that the diseased heart fails by means of its sheer mechanical inability." Certain it is that many a case of heart trouble in which the heart is diseased is best treated by leaving the heart alone and allaying the alarms of the patient. Our schools have become obsessed by obviously material pathology. Their eyes are fixed too intently upon the gross lesions demonstrable after a fatal issue. The study of human nature finds little place in the medical curriculum, and without it both physicians and surgeons, but more particularly physicians, are inadequately qualified for the practice of their profession.

